# A retrospective cohort study of monthly rifampicin, ofloxacin and minocycline in the management of leprosy at the Hospital for Tropical Diseases, London, United Kingdom

**DOI:** 10.1371/journal.pntd.0012699

**Published:** 2024-12-09

**Authors:** Priyanka Sivakumaran, Barbara de Barros, Vivianne Lopes Antonio Dias, Diana N. Lockwood, Stephen L. Walker

**Affiliations:** 1 Royal London Hospital, London, United Kingdom; 2 Hospital for Tropical Diseases, University College London Hospitals NHS Foundation Trust, London, United Kingdom; 3 Faculty of Infectious and Tropical Diseases, London School of Hygiene and Tropical Medicine, London, United Kingdom; 4 Universidade Federal Fluminense, Rio de Janeiro, Brazil; 5 Department of Dermatology, University College London Hospitals NHS Foundation Trust, London, United Kingdom; University of Oxford, UNITED KINGDOM OF GREAT BRITAIN AND NORTHERN IRELAND

## Abstract

**Introduction:**

The World Health Organization (WHO) recommends rifampicin, dapsone and clofazimine multi-drug therapy (MDT) for the treatment of leprosy. Severe adverse effects include dapsone hypersensitivity syndrome, skin pigmentation, haemolytic anaemia, and hepatitis. At the Hospital for Tropical Diseases (HTD), London, United Kingdom monthly rifampicin, ofloxacin and minocycline (mROM) is used as first line treatment for leprosy.

**Objectives:**

To determine the clinical outcomes and experiences of individuals treated with mROM.

**Methods:**

A retrospective study of individuals with leprosy who were prescribed mROM at HTD was conducted. Demographic and clinical data were collected on outcomes including relapses, leprosy reactions, bacterial index (BI) and adverse effects. Individuals were interviewed using a semi-structured questionnaire to understand their experiences of mROM.

**Results:**

29 individuals were identified and 20 interviewed. 26 (89.7%) individuals completed monthly mROM. 9 (31%) had switched from WHO MDT to mROM (five of whom (55.6%) were interviewed). BI reduced significantly following mROM treatment (p = 0.04). 17 individuals (58.6%) experienced a leprosy reaction. One of the 29 (3.4%) relapsed. The relapse rate was 9.5/1000 person years. 49 reports of adverse effects were either mild or moderate. The most frequent adverse effect (14/49) reported was orange discolouration of urine. No adverse effect required hospitalisation or discontinuation of mROM. Most individuals reported that skin lesions improved by the time they had completed mROM.

**Conclusions:**

In this small study in a non-endemic setting mROM was safe, effective and acceptable. mROM therapy is associated with improvement in skin lesions, decline in bacterial index and acceptable adverse effects. Larger, prospective, randomised studies are needed to determine whether relapse rates with mROM are equivalent or better than WHO MDT and to provide robust data on the seemingly better adverse effect profile of mROM.

## Author summary

Leprosy is a chronic disabling infectious disease caused by *Mycobacterium leprae* and *Mycobacterium lepromatosis*. Anti-bacterial treatment with rifampicin, dapsone and clofazimine recommended by the World Health Organization (WHO) is effective but associated with significant adverse effects.

We report the effectiveness of monthly rifampicin, ofloxacin and minocycline in 29 individuals which was not associated with severe adverse effects. The monthly antimicrobial regime can be directly observed during routine clinical assessment reducing issues of adherence.

Alternative antibacterial regimes for leprosy, including newer anti-mycobacterial agents and monthly dosing, should be compared to the WHO recommended standard of care in well-designed randomised controlled trials to assess efficacy and safety.

## Background

Leprosy is caused by *Mycobacterium leprae* and *Mycobacterium lepromatosis* and predominantly affects the skin and peripheral nerves [1]. Leprosy is a highly stigmatised disease due largely to the disability and permanent, visible physical impairments due to the neuropathy and immune-mediated inflammatory leprosy reactions. In 2022, 174 087 new cases of leprosy were reported to WHO by 128 countries and 95% of these cases were reported by 23 countries [2]. Leprosy is designated a neglected tropical disease (NTD).

The World Health Organization (WHO) recommends anti-microbial multi-drug therapy (MDT) to treat *M*. *leprae* and *M*, *lepromatosis* infection with three drugs: rifampicin, dapsone and clofazimine. WHO MDT is provided free of charge pre-packaged in 28-day blister packs (for adults and children older than 10 years) and distributed to governments by WHO. Each adult blister pack contains a monthly dose of rifampicin 600 mg, clofazimine 300 mg and dapsone 100 mg. Dapsone 100 mg and clofazimine 50mg are taken daily on the other 27 days. WHO recommends six months MDT for individuals with paucibacillary (PB) leprosy and 12 months for those with multibacillary (MB) leprosy [3].

WHO-recommended MDT is effective and was introduced in 1982 to standardise treatment and overcome the widespread problem of dapsone resistance following its use as monotherapy. MDT has high cure rates of 99% [4]. The reported relapse rate for participants taking 12 months MB MDT was reported to be 3.1% at 10 years in the Uniform Multidrug Therapy Regimen for Leprosy Patients (U-MDT) trial from Brazil [5,6]. It is estimated over 18 million people have been treated with MDT [7].

MDT is associated with adverse effects which can affect adherence, mental wellbeing and in some cases be fatal [8]. The Global Leprosy Programme has recognised the need for improved pharmacovigilance. In a study from Brazil 70.7% of adverse effects associated with MDT were attributed to dapsone [9]. Dapsone has dose-dependent adverse effects such as methaemoglobinaemia, bone marrow aplasia and haemolytic anaemia [8]. Dapsone Hypersensitivity Syndrome (DHS) has increased frequency in individuals with HLA-B*13: 01 genotype associated and is associated with significant mortality [10]. In a systematic study, a total of eleven prospective and retrospective studies were used to calculate an estimated incidence of DHS as 1.22% (range 0.82% to 3.0%) [8,10].

Clofazimine is a red dye and the commonest adverse effect is skin discoloration, ranging from red to black. The pigmentation usually fades within 12 months of stopping clofazimine, although traces of discoloration may remain for up to one year [11]. The skin discoloration associated with clofazimine is distressing, stigmatising and discloses the diagnosis to those who are aware of this adverse effect [12]. A study in India revealed 9.8% MB patients stopped taking their WHO-recommended MDT due to clofazimine pigmentation [13]. Clofazimine also causes a marked ichthyosis of the skin. A monthly dose of rifampicin as used in MDT causes a brief discolouration of body fluids for up to 48 hours after administration and very rarely hepatitis has been reported [13].

The recent WHO strategic framework for integrated control and management of skin-related NTDs highlighted anti-microbial therapy for leprosy as a research gap in part because of the adverse effects associated with WHO-recommended MDT [14]. The current Global Leprosy Strategy 2021–2030 lists “More effective drugs or drug combinations, or shorter regimes, to treat leprosy” as a research area of “key importance” [7].

The Hospital for Tropical Diseases (HTD) in London operates the only dedicated Leprosy Clinic in the United Kingdom [15]. Leprosy in the UK is a rare disease and is often diagnosed late. There has been no reported autochthonous transmission since the 1954 [16]. Individuals with leprosy managed at the HTD were treated with WHO-recommended MDT unless adverse effects occurred in which case, they were switched to monthly rifampicin 600 mg, ofloxacin 400 mg and minocycline 100 mg (mROM). In 2016 it was decided to use mROM as first-line anti-microbial therapy for all individuals diagnosed with leprosy.

Rifampicin resistance may arise if clofazimine or dapsone are not taken regularly, a suggested alternative is mROM [17], which has been studied in a small number of controlled trials. In a sixth-month course of WHO MDT versus mROM in 268 individuals with paucibacillary (PB) leprosy in India, cure and relapse rates were similar in both groups [18]. A small study of 21 individuals with borderline lepromatous (BL) leprosy and lepromatous leprosy showed mROM for 24 months was as effective as MDT in clinical and bacteriological improvement [19]

We wished to determine the clinical outcomes and experiences of individuals treated with mROM at the HTD.

## Methodology

### Ethics statement

Ethical approval was granted by the London School of Hygiene and Tropical Medicine MSc Research Ethics Committee (27207) and the University College London Hospitals NHS Foundation Trust in accordance with the institutional policy.

### Study design

We performed a retrospective cohort study to determine the clinical outcomes of individuals with leprosy treated with mROM at the HTD and any adverse effects attributed to mROM. Data were collected on the occurrence of leprosy reactions: Type 1 reactions (T1R) and erythema nodosum leprosum (ENL). T1Rs were treated with oral prednisolone. ENL was treated with oral prednisolone and/or thalidomide.

Individuals who were included in the retrospective analysis and still attending the Leprosy Clinic were invited to participate in a semi-structured interview about their experiences of taking anti-microbial medication including their recall of adverse effects. Translator services were used if needed.

The severity of reported adverse effects on activity of daily life was graded using the Common Terminology Criteria for Adverse Events [20].

### Study setting

The HTD is a national referral centre in the UK for tropical and infectious diseases and has a dedicated Leprosy Clinic.

### Data collection and study population

All individuals with a confirmed diagnosis of leprosy who attended the Leprosy Clinic and received mROM treatment between 1^st^ January 2008 and 31^st^ December 2021 were eligible. Data were collected from medical records in a standardised data collection form.

Our previously reported case definitions for the diagnosis of leprosy and leprosy reactions were used for the current study [21]. The diagnosis of leprosy and leprosy reaction was made based on the cardinal signs of leprosy or other clinical signs supported by histopathology.

The semi-structured questionnaire (S1 Interviewee questionnaire) was completed by a member of the research team (PS) as part of a face-to-face or telephone interview between 1^st^ June-31^st^ August 2022. All individuals who participated in the interviews gave written informed consent.

### Data management and analysis

All data were anonymised and entered in Excel (S1 Data). Clinical data were analysed with descriptive statistics. Paired t-test was used to analyse change in bacterial index (BI). A p-value of <0.05 was considered statistically significant.

Adverse effects or symptoms reported by interviewees were attributed to the most likely cause based on the adverse effect profile of medications (individual components of mROM, prednisolone or thalidomide), the reported timing of onset and the management instituted. Where an adverse effect could be attributed to two or more components of mROM then mROM was listed as the most likely cause rather than individual components.

## Results

29 individuals received mROM and 26 (89.7%) completed treatment with mROM. 20 individuals were interviewed.

The demographic and clinical data are summarised in Table 1. The majority of individuals were male (69%), and most individuals had positive slit-skin smears including 12 (41.3%) who had a mean BI of 4 or more. Leprosy reactions occurred in 17 (48.7%) of individuals.

Four individuals had hypertension, four had diabetes mellitus, and one had HIV infection. No individual was on a medication known to interact with rifampicin or ofloxacin or minocycline.

**Table 1:** The demographics and clinical data of the 29 patients.

Characteristic		Number (%) [Total = 29]
**Sex**	**Female**	9 (31)
	**Male**	20 (69)
**Age in years at diagnosis (median)**	39 years (IQR: 31 to 50 years)	
**Ridley-Jopling classification**	**TT**	4 (13.8)
	**BT**	6 (20.7)
	**BB**	0 (0)
	**BL**	4 (13.8)
	**LL**	14 (48.3)
	**PNL**	1 (3.4)
**Mean Bacterial Index at diagnosis**	**0**	9 (31)
	**0.1–0.99**	0 (0)
	**1–1.99**	3 (10.3)
	**2–2.99**	1 (3.4)
	**3–3.99**	0 (0)
	**4.4.99**	3 (10.3)
	**5–5.99**	8 (27.6)
	**6**	1 (3.4)
	**No data**	4 (13.8)
**WHO Disability Grade at diagnosis**	**0**	9 (31)
	**1**	12 (41.3)
	**2**	8(27.6)
**Anti-microbial treatment at diagnosis**	**WHO MDT**	9 (31)
	**mROM**	20 (69)
**Duration of mROM (months)**	**6**	7 (24.1)
	**12**	13 (44.8)
	**24**	2 (6.9)
	**Other**	7 (24.1)
**Follow-up status at time of data collection**	**Continues to attend clinic**	23 (79.3)
	**Discharged**	4 (13.8)
	**Deceased**	2 (6.9)
**Leprosy reactions experienced up to data collection**	**None**	12 (41.3)
	**Type 1 reaction**	5 (17.2)
	**ENL**	9 (31)
	**Both**	3 (10.3)

TT, tuberculoid leprosy; BT, borderline tuberculoid; BB, mid-borderline; BL, borderline lepromatous; LL, lepromatous leprosy; PNL, pure neural leprosy; WHO MDT, World Health Organization multi-drug therapy; mROM, monthly rifampicin ofloxacin and minocycline; ENL, erythema nodosum leprosum.

Nine (31%) individuals were switched to mROM from WHO MDT. The reasons for the switch to mROM recorded in the patient records were as follows: five individuals were switched due to adverse effects of clofazimine, four for hyperpigmentation and one due to hyperpigmentation and ichthyosis. One individual stopped WHO MDT because of dapsone induced haemolytic anaemia and another due to dapsone supply issues. Two individuals did not adhere to daily treatment and so were offered mROM.

Five of the 29 patients were lost to follow-up. The total follow-up time was 96 years with a median of 3.10 years (IQR: 1.32 to 4.84 years). All individuals had a satisfactory response to anti-microbial treatment. One individual relapsed two years after completing 12 doses of mROM (9.5/1000 person years).

Most individuals received a 6-month or 12-month course of mROM. Variations in duration was due to switching from WHO MDT and completing the course duration with mROM.

Twenty-five individuals had a baseline BI, the mean BI was 3 (SD 2.31). Nine had at least one repeat biopsy or smear. The mean time between the first BI and the last recorded BI was 39.2 months (SD: 21.0 months). Of these individuals the mean BI at diagnosis was 4.00 ±1.94 and the mean of the last recorded BI was 2.69±3.72. This difference was statistically significant (p = 0.04).

Seventeen individuals had leprosy reactions (58.6%). Nine (52.9%) had ENL, five (29.5%) had T1R, three (17.6%) individuals had both T1R and ENL. All ENL and T1R required corticosteroids. Individuals with ENL were switched to thalidomide, 5 of the 17 individuals with reactions switched from WHO MDT. The odds of having a leprosy reaction at any time for those who received mROM compared to those who switched from WHO MDT to mROM was 1.2 (95% CI:0.25 to 5.9) p = 0.822

The mean time from completing monthly mROM to interviews being conducted was 2 years (IQR: 0 to 3 years). 18 of 20 (90%) interviewees reported 49 adverse effects (Table 2).

**Table 2:** Forty-nine adverse effects reported by 18 of 20 interviewees.

Putative cause of adverse effect	Reported adverse effect	Number (%) [Total 49]
Rifampicin	Orange urine	14 (28.6)
mROM or leprosy reaction	Myalgia	4 (8.2)
	Joint pain or stiffness	4 (8.2)
mROM	Nausea	3 (6.1)
	Dizziness	2 (4.1)
	Breathlessness	1 (2)
	Bruising	1 (2)
	Candidiasis	1 (2)
	Dermatitis	1 (2)
	Headache	1 (2)
	Menorrhagia	1 (2)
	Night sweats	1 (2)
	Tinea corporis	1 (2)
	Vomiting	1 (2)
	Yellow sclera	1 (2)
mROM or leprosy reaction treatment	Low mood	2 (4.1)
Leprosy reaction treatment	Insomnia	2 (4.1)
	Loss of concentration	1 (2)
Leprosy reaction	Oedema	2 (4.1)
	Fever	1 (2)
Leprosy reaction or leprosy reaction treatment	Fatigue	1 (2)
Leprosy or leprosy reaction	Paraesthesia	2 (4.1)
Leprosy	Epistaxis	1 (2)

All adverse effects were mild or moderate based on Common Terminology Criteria for Adverse Events. Orange urine due to rifampicin was the most common reported adverse effect of mROM; 14 out of 49 (28.6%). Most adverse effects were non-specific and could be attributed to one or more of mROM, leprosy reactions, corticosteroids or thalidomide, and leprosy per se. mROM was thought to be the cause of a further 15 (30.6%) adverse effects. mROM and leprosy reaction or mROM and leprosy reaction treatment were thought to be responsible for 10 (20.4%) reported adverse effects. The remaining 10 (20.4%) were attributed to leprosy, leprosy reactions or leprosy reaction treatment. No adverse effects needed hospitalisation and no individual stopped mROM due to adverse effects. 50% of adverse effects occurred within 24 hours of starting mROM.

## Discussion

We report good clinical outcome in a cohort of 29 individuals who received mROM for leprosy and were followed for a median period of 3.1 years. The proportion of individuals who experienced a leprosy reaction was 58.6% which is consistent with leprosy reaction rates of approximately 65% at three years in a large Brazilian cohort of individuals with MB leprosy treated with WHO MDT containing rifampicin, clofazimine and dapsone for six or 12 months [5]. Pre- and post-mROM treatment BI data were available in 31% and indicated a significant decline in BI. One individual relapsed following mROM. Interestingly this was their second relapse. mROM having been chosen to treat their lepromatous leprosy in part because they had relapsed following treatment with rifampicin and dapsone for borderline tuberculoid leprosy several years earlier.

Ten individuals (eight of whom had lepromatous leprosy) were successfully treated with 12 or 24 months of monthly rifampicin, moxifloxacin and minocycline in the United States [21]. One individual experienced transient mild elevation of liver transaminases and two had “minor gastrointestinal side effects”. Another study by Faust et al. reported mROM used to treat three patients. Two with tuberculoid leprosy received six doses and one with BL leprosy received 24 doses. There were no adverse events requiring discontinuation of treatment [22].

All three anti-bacterial drugs in mROM are bactericidal for *M*. *leprae* whereas in WHO MDT dapsone is bacteriostatic and clofazimine only weakly bactericidal. A single dose of mROM was shown to exhibit ≥97.5% bactericidal activity in nine of 10 individuals with lepromatous leprosy [17]. Mouse studies indicate that a single dose of mROM has less bactericidal activity than one month of WHO MDT [17] but the authors felt that it would be reasonable to compare several monthly doses of mROM with WHO MDT used for a similar duration.

Our cohort interviewees reported 49 potential adverse effects of which 79.6% were attributable to a component of mROM. No severe adverse effects were reported by the interviewees or documented in their medical records. A recent retrospective Brazilian study of the medical records of 443 individuals with leprosy showed that the risk of adverse effects was significantly greater in those who were treated with WHO MDT than those treated with mROM for 12 or 24 months [23]. Thirty-four of 247 (13.8%) individuals treated with mROM experienced an adverse reaction but there were no reports of rifampicin associated discolouration of urine which accounted for 35.9% of mROM related adverse effects in our interviewees.

Interestingly tendinopathies did not occur in our cohort nor the Brazilian study. Fluoroquinolones (including ofloxacin) are the subject of prescribing restrictions by regulatory agencies in the United States, United Kingdom and European Union [24–26] because of the increased risk of tendinopathy and other serious adverse effects including aortic dissection. A systematic review and meta-analysis by Alves et al. described an increased risk of tendon disorders from fifteen studies of individuals treated with fluoroquinolones [27]. Older age (≥ 60 years) and concomitant use of corticosteroids, particularly important in the context of leprosy reactions, were additional risk factors. Ofloxacin was associated with a higher rate of tendon disorders compared to ciprofloxacin and levofloxacin [28] in a more recent meta-analysis. Neither of these systematic reviews was able to comment in detail on the dose and duration of fluoroquinolone used but it would be reasonable to assume they were not used as a single monthly dose although a single dose of ofloxacin 400 mg is indicated for gonococcal urethritis and cervicitis due to susceptible *Neisseria gonorrhoeae* [29].

Our study has several limitations. The small number of participants and retrospective nature mean data should be interpreted with caution and the results of interviews often conducted months or years after completion of mROM may have been subject to recall bias and difficulty in attributing causality for adverse effects reliably. However, the use of interviews allows the voice of affected individuals to contribute to the understanding of the adverse effect profile of mROM which may not be captured by clinician documentation in medical records.

Our experience of the clinical effectiveness of mROM and the associated mild adverse effects has led us to use this combination first-line for the management of leprosy. The anti-microbials are usually taken during the monthly clinic attendance when nerve function assessment is undertaken. Treatment adherence is assured and “pill burden” reduced. Recently moxifloxacin has replaced ofloxacin because of the greater bactericidal activity of the former [30]. Robust clinical trials of alternative regimes to WHO MDT are required to provide evidence for efficacious (and potentially shorter) antibacterial regimes with reduced risk of severe adverse effects for people with leprosy. There is sufficient evidence to suggest that one intervention arm should be a monthly regime of rifampicin, moxifloxacin and minocycline.

## Supporting information

S1 STROBE checklist STROBE Statement—checklist of items that should be included in reports of observational studies. (DOC)

S1 Interviewee questionnaire Blank questionnaire sheet used to collect data during patient interviews. (DOCX)

S1 Data Clinical data on 29 patients with leprosy. (XLSM)
