# Supplementary material for: A retrospective cohort study of monthly rifampicin, ofloxacin and minocycline in the management of leprosy at the Hospital for Tropical Diseases, London, United Kingdom
Source: PLoS Negl Trop Dis. 2024 Dec 9;18(12):e0012699. doi: 10.1371/journal.pntd.0012699 (PMC11658689; doi:10.1371/journal.pntd.0012699)
Supplement: S1 STROBE checklist — (DOC) [file pntd.0012699.s001.doc]

STROBE Statement—checklist of items that should be included in reports of observational studies

|  | Item No | Recommendation |  |
| --- | --- | --- | --- |
| **Title and abstract** | 1 | (*a*) Indicate the study’s design with a commonly used term in the title or the abstract | The term retrospective is used in the title and abstract in the methods section. |
|  |  | (*b*) Provide in the abstract an informative and balanced summary of what was done and what was found | Contained in the methods and results |
| Introduction | | |  |
| Background/rationale | 2 | Explain the scientific background and rationale for the investigation being reported | Background pages 4-6 |
| Objectives | 3 | State specific objectives, including any prespecified hypotheses | Page 6 “We wished to determine the clinical outcomes and experiences of individuals treated with mROM at the HTD.” |
| Methods | | |  |
| Study design | 4 | Present key elements of study design early in the paper | Study design section of methods pages 6-7 |
| Setting | 5 | Describe the setting, locations, and relevant dates, including periods of recruitment, exposure, follow-up, and data collection | Study design and data collection settings pages 6-7 |
| Participants | 6 | (*a*) *Cohort study*—Give the eligibility criteria, and the sources and methods of selection of participants. Describe methods of follow-up  *Case-control study*—Give the eligibility criteria, and the sources and methods of case ascertainment and control selection. Give the rationale for the choice of cases and controls  *Cross-sectional study*—Give the eligibility criteria, and the sources and methods of selection of participants | “All individuals with a confirmed diagnosis of leprosy who attended the Leprosy Clinic and received mROM treatment between 1st January 2008 and 31st December 2021 were eligible.” On page 7 in Data collection and study population section. |
|  |  | (*b*) *Cohort study*—For matched studies, give matching criteria and number of exposed and unexposed  *Case-control study*—For matched studies, give matching criteria and the number of controls per case |  |
| Variables | 7 | Clearly define all outcomes, exposures, predictors, potential confounders, and effect modifiers. Give diagnostic criteria, if applicable | Clinical outcomes including bacterial index and reported adverse effects pages 6 and 8 |
| Data sources/ measurement | 8* | For each variable of interest, give sources of data and details of methods of assessment (measurement). Describe comparability of assessment methods if there is more than one group | Data source was the clinical record and participant reported symptoms described on page 7. |
| Bias | 9 | Describe any efforts to address potential sources of bias | None |
| Study size | 10 | Explain how the study size was arrived at | All individuals meeting the inclusion criteria. |
| Quantitative variables | 11 | Explain how quantitative variables were handled in the analyses. If applicable, describe which groupings were chosen and why | Data management and analysis. Page 8 |
| Statistical methods | 12 | (*a*) Describe all statistical methods, including those used to control for confounding | Data management and analysis. Page 8 |
|  |  | (*b*) Describe any methods used to examine subgroups and interactions | None |
|  |  | (*c*) Explain how missing data were addressed | None |
|  |  | (*d*) *Cohort study*—If applicable, explain how loss to follow-up was addressed  *Case-control study*—If applicable, explain how matching of cases and controls was addressed  *Cross-sectional study*—If applicable, describe analytical methods taking account of sampling strategy |  |
|  |  | (*e*) Describe any sensitivity analyses | None |

| Results | | |  |
| --- | --- | --- | --- |
| Participants | 13* | (a) Report numbers of individuals at each stage of study—eg numbers potentially eligible, examined for eligibility, confirmed eligible, included in the study, completing follow-up, and analysed | Results page 8 “29 individuals received mROM and 26 (89.7%) completed treatment with mROM. 20 individuals were interviewed.” |
|  |  | (b) Give reasons for non-participation at each stage | Loss to follow up. |
|  |  | (c) Consider use of a flow diagram | Not included. |
| Descriptive data | 14* | (a) Give characteristics of study participants (eg demographic, clinical, social) and information on exposures and potential confounders | Table 1 Page 9 |
|  |  | (b) Indicate number of participants with missing data for each variable of interest | Table 1 Page 9 |
|  |  | (c) *Cohort study*—Summarise follow-up time (eg, average and total amount) | Page 10 |
| Outcome data | 15* | *Cohort study*—Report numbers of outcome events or summary measures over time | Results pages 10 and 11 |
|  |  | *Case-control study—*Report numbers in each exposure category, or summary measures of exposure |  |
|  |  | *Cross-sectional study—*Report numbers of outcome events or summary measures |  |
| Main results | 16 | (*a*) Give unadjusted estimates and, if applicable, confounder-adjusted estimates and their precision (eg, 95% confidence interval). Make clear which confounders were adjusted for and why they were included | Results pages 10 and 11 |
|  |  | (*b*) Report category boundaries when continuous variables were categorized | Results pages 10 and 11 |
|  |  | (*c*) If relevant, consider translating estimates of relative risk into absolute risk for a meaningful time period |  |
| Other analyses | 17 | Report other analyses done—eg analyses of subgroups and interactions, and sensitivity analyses | Not applicable |
| Discussion | | |  |
| Key results | 18 | Summarise key results with reference to study objectives | Discussion pages 12 and 13 |
| Limitations | 19 | Discuss limitations of the study, taking into account sources of potential bias or imprecision. Discuss both direction and magnitude of any potential bias | Discussion pages 12 and 13 |
| Interpretation | 20 | Give a cautious overall interpretation of results considering objectives, limitations, multiplicity of analyses, results from similar studies, and other relevant evidence | Discussion pages 12 and 13 |
| Generalisability | 21 | Discuss the generalisability (external validity) of the study results | Discussion pages 12 and 13 |
| Other information | | |  |
| Funding | 22 | Give the source of funding and the role of the funders for the present study and, if applicable, for the original study on which the present article is based | Funding statement 14 |

*Give information separately for cases and controls in case-control studies and, if applicable, for exposed and unexposed groups in cohort and cross-sectional studies.

**Note:** An Explanation and Elaboration article discusses each checklist item and gives methodological background and published examples of transparent reporting. The STROBE checklist is best used in conjunction with this article (freely available on the Web sites of PLoS Medicine at http://www.plosmedicine.org/, Annals of Internal Medicine at http://www.annals.org/, and Epidemiology at http://www.epidem.com/). Information on the STROBE Initiative is available at www.strobe-statement.org.
