# Supplementary material for: A retrospective cohort study of monthly rifampicin, ofloxacin and minocycline in the management of leprosy at the Hospital for Tropical Diseases, London, United Kingdom
Source: PLoS Negl Trop Dis. 2024 Dec 9;18(12):e0012699. doi: 10.1371/journal.pntd.0012699 (PMC11658689; doi:10.1371/journal.pntd.0012699)
Supplement: S1 Interviewee questionnaire — (DOCX) [file pntd.0012699.s002.docx]

***Thank you for taking part in this questionnaire. This questionnaire is looking into any reactions, if any, you have experienced while being treated with rifampicin, ofloxacin and minocycline for leprosy. This questionnaire is anonymous and will not affect your care in any way.***

1. Questionnaire Number: ______
2. Hospital ID: ________________
3. Do you remember taking medication for treatment of leprosy?

Yes **▢**

No **▢**

1. Do you remember the names of the medications you were on?

_______________________________________________________________________________________________________________________________________________________________________________________________________________

1. Was this your only treatment or have you had treatment before for leprosy?

Yes **▢ (**Please go to question 10)

No **▢ (**Please go to question 9)

1. What was the other treatment? How did you feel about the other treatment?

_______________________________________________________________________________________________________________________________________________________________________________________________________________

1. Do you know why you were changed to another treatment?

_______________________________________________________________________________________________________________________________________________________________________________________________________________

1. How did you feel about starting rifampicin, ofloxacin and minocycline (ROM)?

_______________________________________________________________________________________________________________________________________________________________________________________________________________

1. For your ROM treatment, did you take the medicine under direct supervision, how did you feel about that?

_______________________________________________________________________________________________________________________________________________________________________________________________________________

1. While taking ROM, did you experience any heart or circulation symptoms such as:

Bleeding or bruising ▢ Unusual heartbeat ▢

Other________________________________________________________________

Can you explain more about the symptom(s):

____________________________________________________________________

1. While taking ROM, did you experience any breathing symptoms such as:

Breathlessness ▢ Wheeze ▢ Throat Swelling ▢ Cough ▢

Other________________________________________________________________

Can you explain more about the symptom(s):

____________________________________________________________________

1. While taking ROM, did you experience any digestive symptoms such as:

Loose stools ▢ Not feeling like eating ▢ Vomiting ▢

Nausea ▢ Tummy pains ▢ Constipation ▢

Difficulty swallowing ▢ Heartburn ▢

Other________________________________________________________________

Can you explain more about the symptom(s):

____________________________________________________________________

1. While taking ROM, did you experience any of the following brain or nervous system symptoms:

Seeing or hearing anything unusual ▢ Dizziness ▢

Change in eyesight ▢ Eye irritation ▢

Headache ▢ Ringing in ears ▢ Change in hearing ▢ Anxiety ▢ Change in taste ▢ Change in sleep ▢

Low mood ▢ Nightmares ▢ Numbness ▢

Abnormal movements e.g., twitching, difficulty walking ▢

Other________________________________________________________________

Can you explain more about the symptom(s):

____________________________________________________________________

1. While taking ROM, did you experience any of the muscle or joint symptoms:

Weakness ▢ Muscle pain ▢ Joint pain ▢

Bone pain ▢ Swelling/ pain in tendon ▢ Tendon breaking ▢

Hair loss ▢ Painful and swollen tongue ▢

Other________________________________________________________________

Can you explain more about the symptom(s):

____________________________________________________________________

1. While taking ROM, did you experience any of these skin or hair symptoms:

Rash ▢ Yellow skin/ eyes ▢ Darkening of skin ▢

Red colour urine/sweat/tears ▢ Hot flushes ▢

Increased sweating ▢ Hair loss ▢

Changes in colour of white part of eyes ▢

Skin/nail colour change to blue-grey ▢

Tear colour change ▢ Teeth colour change▢

Other________________________________________________________________

Can you explain more about the symptom(s):

____________________________________________________________________

1. While taking ROM, did you experience any of these symptoms:

Fever ▢ Frequent infections▢ Flu like symptoms ▢

Fungal infections ▢

Other________________________________________________________________

Can you explain more about the symptom(s):

____________________________________________________________________

1. While taking ROM, did you experience any of these reproductive symptoms?

Menstrual changes ▢ Please explain:_______________________________

Other________________________________________________________________

Can you explain more about the symptom(s):

____________________________________________________________________

1. Anything else you have experienced when you were on ROM?

_______________________________________________________________________________________________________________________________________________________________________________________________________________

1. Please explain in a bit more detail about the reaction you experienced above:

What reaction(s) was it: _______________________________________________________________

How soon after taking ROM did it happen __________ days/months

How long it did it last for?___________ days/months or ongoing ▢

Where you given anything by your doctor to help with this reaction?

Yes ▢ What was this? ____________________________________

No ▢

1. With the reaction(s) you mentioned above, was the reaction:
   - - 1. Mild – where you needed nothing to help with the reaction ▢
       2. Mild-moderate, the reaction limited some of your daily activities. No or little medical help needed. ▢
       3. Severe or significant enough that assistance is was needed for daily activities or medical help was needed. And you may even had to attend hospital ▢
       4. Extremely limited your daily activity, life threatening. Significant medical help needed with hospital admission ▢
2. Did you ever stop your ROM treatment?

Yes ▢ (Please go to question 25)

No ▢ (Please go to question 27)

1. Why did you stop taking ROM?

_______________________________________________________________________________________________________________________________________________________________________________________________________________

1. Did you re-start taking ROM?

Yes ▢ Why? _________________________________________________________

No ▢

1. If you completed taking you treatment, how do you feel about finishing ROM treatment?

_______________________________________________________________________________________________________________________________________________________________________________________________________________

1. Do you have any comments you want to share about your treatment with ROM?

_______________________________________________________________________________________________________________________________________________________________________________________________________________

***Thank you for taking part in this questionnaire. Your answers will be kept confidential and anonymous unless there is a risk to your health, then your doctor will be informed. If you have any further questions, please contact Dr Priyanka Sivakumaran via email: p.sivakumaran@nhs.net***
